# Crohn’s and Colitis Foundation of America Partners Patient-Powered Research Network: Patient Perspectives on Facilitators and Barriers to Building an Impactful Patient-Powered Research Network

**DOI:** 10.1097/MLR.0000000000000771

**Published:** 2018-09-13

**Authors:** Arlene E. Chung, Maihan B. Vu, Kelly Myers, Jessica Burris, Michael D. Kappelman

**Affiliations:** * Department of Medicine, Division of General Internal Medicine and Clinical Epidemiology; † Department of Pediatrics, Division of General Pediatrics and Adolescent Medicine; ‡ Program on Health and Clinical Informatics, University of North Carolina (UNC) at Chapel Hill, Chapel Hill School of Medicine; § Carolina Health Informatics Program, UNC Chapel Hill; ∥ Department of Health Behavior, Gillings School of Global Public Health, Center for Health Promotion and Disease Prevention; ¶ Lineberger Comprehensive Cancer Center, University of North Carolina at Chapel Hill, Chapel Hill, NC; # Atomo Health Inc., Austin, TX; ** Vanderbilt University School of Medicine, Nashville, TN; †† Division of Pediatric Gastroenterology, Center for Gastrointestinal Biology and Disease, UNC Chapel Hill School of Medicine, Chapel Hill, NC

**Keywords:** patient engagement, patient-generated health data, PGHD, wearables, patient-powered research, patient-centered care, patient-reported outcomes, PROs, PPRN, patient-powered research networks, PCORI

## Abstract

**Background:**

To build a Patient-Powered Research Networks (PPRN) that prioritizes the needs of its members who have inflammatory bowel diseases (IBD), we sought to better understand patients’ preferences for what are the essential features that will facilitate and sustain engagement.

**Methods:**

We conducted a two-phase qualitative study. Seven focus groups involving 62 participants with IBD were conducted (phase 1). Focus group results informed the phase 2 cognitive interviews, which included 13 phone interviews. Topics included experiences with IBD and research, PPRN engagement, patient-generated health data, and resources/tools to facilitate self-management. All focus groups and interviews were digitally recorded, transcribed verbatim, and analyzed in ATLAS.ti 7.5. Thematic categories were derived from the data, and codes were grouped into emergent themes and relationships.

**Results:**

Four major themes emerged through inductive coding: (1) the impact of knowing; (2) participation barriers and challenges; (3) engagement and collaboration; and (4) customizable patient portal features/functionalities. Participants were motivated to participate in the PPRN because the knowledge gained from research studies would benefit both society and the individual. Main concerns included credibility of online resources, pharmaceutical industry profiting from their data, data security, and participation expectations. Participants wanted a true and equal partnership in every phase of building a PPRN. Participants felt it was important to have access to personal health records and be able to track health status and symptoms.

**Conclusion:**

Partnering with participants throughout PPRN development was critical to understanding the needs and preferences of patients with IBDs and for shaping engagement strategies and the portal’s design.

## Abstract 2

Supplemental Digital Content is available in the text.

Patient-Powered Research Networks (PPRNs) have the potential to improve health behaviors and outcomes,^1^,^2^ yet engaging patients to participate is a universal challenge. Strategies for engagement and collaboration in a patient-centered PPRN are complex and require thoughtful consideration of multiple factors. In particular, it is important to reflect the diverse nature of real-world patients and their unique set of circumstances, both physical and social. PPRNs are faced with multiple issues including engaging patients throughout the life cycle of research, securely sharing data while ensuring privacy, prioritizing the needs of the patient communities, and identifying ways to sustain engagement beyond initial enrollment.^1^,^3^ Online patient communities such as PatientsLikeMe are exemplars for data sharing, and patients have perceived benefits from participation such as improved social support, information about treatments, and contributing toward research to understand the cause and nature of diseases.^4^–^6^ Although many focus on data sharing, few networks involve patients in the entire life cycle of research from the conception of research question to dissemination of results, research prioritization, or allow the contribution of wearable device data so it is critical to understand patient perspectives in designing a new PPRN.

The overarching goal for the Patient-Centered Outcomes Research Institute Crohn’s and Colitis Foundation of America Partners (CCFA) PPRN is to promote both inflammatory bowel diseases (IBD) research and disease management to improve patient-centered outcomes. This effort builds upon a novel Internet cohort of patients with IBD and focuses on patient-reported exposures, wearable device data, health behaviors, and outcomes.^7^

Because PPRNs for IBDs and other chronic conditions are an emerging concept, we aimed to elicit the perceived facilitators and barriers of participating in an IBD-focused PPRN and to better understand patients’ preferences for the design of an online portal that will facilitate and sustain engagement in research as there few PPRN portals nationally. As patient-generated health data from wearable devices, sensors, and smartphone applications, and portals may be new concepts for many patients, participants also reviewed various mockups of the PPRN portal’s user interface to assess preferences for potential features and functionalities.

## METHODS

### Study Design

We conducted a two-phase qualitative study that consisted of: (1) focus group discussions; and (2) cognitive interviews. Focus group discussions were designed to understand participants’ experiences and needs managing their disease, outcomes most important to participants, and ways to make a PPRN most useful. Individual cognitive interviews were then used as a complementary approach to additionally explore and assess patient portal user interface prototypes and ways the portal could help track and manage IBD.

### Sampling and Study Participants

Participants were recruited using several strategies including patient registries, provider invitations, and clinic flyers. We used purposeful sampling^8^ to obtain rich information from subgroups of participants who were geographically and racially diverse with a balance of disease conditions (Crohn’s disease and ulcerative colitis) age, and sex. This involved targeted invitations to registry or clinic patients to recruit for minorities traditionally underrepresented in IBD studies, older patients, and those from rural versus urban locations. Eligibility criteria for both the focus groups and individual interviews included that participants had to be diagnosed with chronic IBDs, aged 18 and older, and English speaking. Additional eligibility for the interviews included that participants had to be a member of CCFA Partners Internet cohort study which has participants across the United States.

### Data Collection

Semistructured interview guides for the focus groups and interviews were developed that consisted of open-ended questions and prompts about attitudes and beliefs about IBD, patient engagement and challenges, and strategies for building a patient-centered research network (Supplementary Table 1, Supplemental Digital Content 1, http://links.lww.com/MLR/B422). The study team and patient partners from the CCFA Partners PPRN patient governance committee participated in interview guide development. All interviews and focus groups were recorded with permission and professionally transcribed verbatim. Focus groups and interviews were conducted by an experienced qualitative researcher with doctoral training in qualitative research methods (M.B.V.). The mean length of focus group discussions was 90 minutes and cognitive interviews lasted 60 minutes. Participants also completed a brief questionnaire to solicit information on demographics, disease condition, technology use, and research participation.

The individual interviews explored similar topics from the focus group discussions with additional questions focused specifically on feedback regarding the portal user interface. Before the interviews, participants received mockups of the PPRN portal user interface via email. During the interview, the participant and interviewer reviewed each mockup and discussed the related questions. This study was conducted from July 2014 to March 2015 and was approved by Institutional Review Board at the University of North Carolina at Chapel Hill (IRB#14-0835).

### Data Analysis

We imported transcripts into ATLAS.ti version 7.5.9 (Scientific Software Development GmbH). Separate codebooks were created to analyze data from the focus group discussions and the cognitive interviews. To enhance trustworthiness, all analyses were conducted independently with no input from sponsors and the lead for analysis had no competing interests (M.B.V.). A senior qualitative researcher (M.B.V.) with extensive experience developed topical codes from the interview guides (eg, disease condition and beliefs) and participants’ words (eg, respect, trust, safety). An inductive approach as described by Strauss and Corbin was used for codes that emerged on rereading (eg, values, social awareness, etc.).^9^ An audit log of decisions was kept along with codes arising from the focus groups and interviews. We then grouped codes into emergent themes and relationships after iterative reading of the transcripts and discussion with the research team. Because the focus groups and interviews evaluated a similar set of topics, we pooled the qualitative data for the final summarization of findings and chose quotes representative of each theme.

## RESULTS

Seven focus group discussions were conducted with 62 participants [5 groups in a Southern state (North Carolina) and 2 groups in a Midwestern state (IL)] with an average of 10 participants per group (range, 5–13). Most participants were middle aged, female (58%), and 73% self-identified as white (Table 1). About 61% were diagnosed with Crohn’s disease and reported an average of 21 years since diagnosis. Almost all had a cellphone, and most had a smartphone, Internet access via cellphones or computers/tablet computers with Internet access. The 7 groups were similar in demographic characteristics, except for age and sex.

**TABLE 1:**
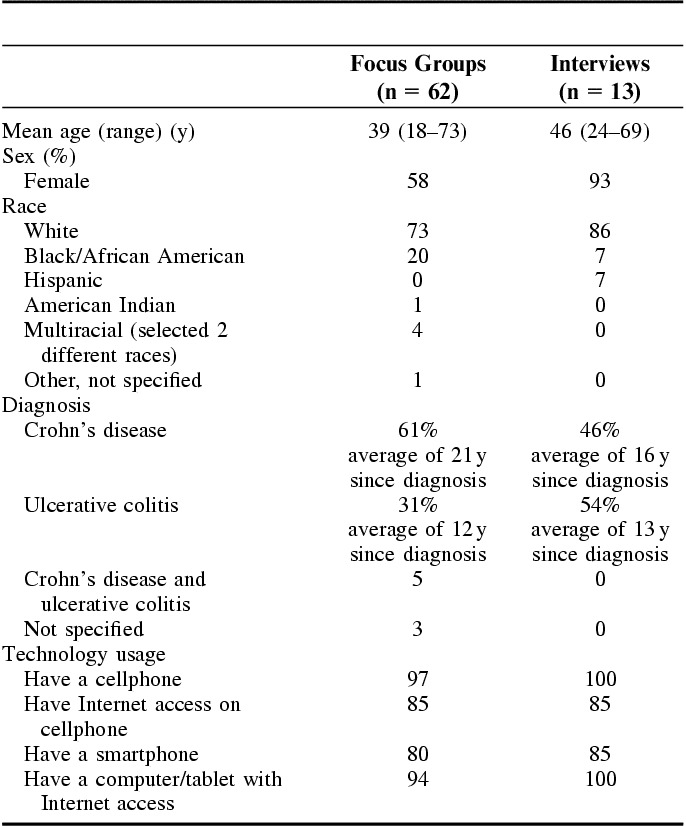
Sample Characteristics

A total of 13 individual interviews were conducted by phone with participants from 11 states. The average patient age was 46 years. Approximately 93% were female and most identified as white. Almost half of the participants had Crohn’s disease and reported an average of 16 years since diagnosis. All had a cellphone and computers/tablets with Internet access. There were no overlapping participants between the focus groups and interviews.

Four predominant themes emerged through analysis of the pooled data from focus groups and interviews: (1) the impact of knowing; (2) participation barriers and challenges; (3) engagement and collaboration; and (4) customizable patient portal features and functionalities. The themes were derived inductively or arose directly from discussions.

### Theme 1: The Impact of Knowing (Information and Knowledge)

Participants said there were multiple motivators for participating in the CCFA PPRN. Perceived personal, social, and health benefits were among the reasons patients desire to participate in research. Participants were more willing to participate knowing that the knowledge gained in research studies would result in both societal and individual level benefits. This included “prevention” of IBD and its complications, finding a cure, and access to new treatment options. Having more information was felt to be important for enhanced IBD awareness and for improvements in understanding and empathy by society.

#### “Prevention” and Cure

Participants explained that although it was “too late” for them, information learned from research might help others to avoid getting IBD (prevention). One participant shared that “it’s not just about me.” There was a sense of giving back and hope that having more information would help uncover ways to stop IBD. Patients with IBDs said they were highly motivated to contribute in any way possible so researchers could find a cure. They also expressed a desire to change their current health status and move beyond “managing” their conditions to having a better quality of life. According to one participant, “A lot of the medical stuff I’ve done before … I want to know, I want to be selfless. I want to help large groups of people … [and] I want the information that you can glean from me—I mean selfishly, it’s probably going to help me one of these days. I can be a part of a research that will help me.”

#### Options for Treatment and Care

Participants were eager to hear about various treatment combinations and options and wanted to learn about others’ experiences with new therapies. They felt having this kind of information led to better health decisions and outcomes. Collectively, participants described themselves as “experimenters” who willingly tried different “cocktails” or mixtures of medications, alternative treatments, and diet/lifestyle changes as needed to control their IBD. They wish that research would give them information about the success rates of these varying treatment approaches.

#### Raising Society’s Level of Understanding and Empathy

Participants felt that making society more aware of IBD was a high priority. They felt having more information would foster an environment that made it comfortable to discuss IBD openly in public. For example, breast cancer was noted as a disease with good public support, high awareness, and sympathy, but many noted that there was less conversation and promotion around IBD. Participants also thought it was very important to garner empathy for participants with IBD at a societal level. They described how participants with IBD shared an understanding and empathy among each other, but the gap was at the societal level. Some participants indicated that despite having this disease for several years, many of their own family members still did not fully understand what IBD was and how it impacted multiple aspects of their lives. According to a participant, “it took my wife about 30 years to understand and I am still not sure she does.”

### Theme 2: Participation Barriers and Challenges

The main concerns about participating in a PPRN included credibility about the type and amount of information online, the pharmaceutical industry profiting from their data, data security/privacy, and time burden and participant expectations. Illustrative quotes are provided in Table 2.

**TABLE 2:**
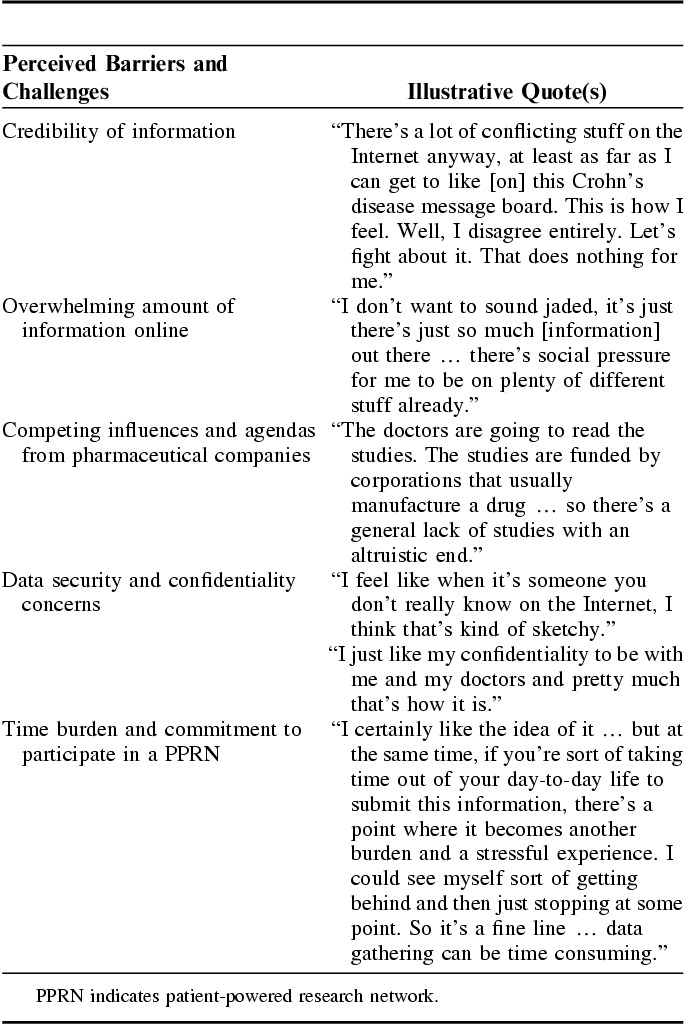
Illustrative Quotes Relating to Themes on Barriers and Challenges to Participate in a PPRN

#### Credibility of Information Source

Participants acknowledged that there is an abundance of online information about IBD and some felt that little was known about the quality and credibility of these sources. If participants were not able to judge where the information originated, whether there were hidden agendas, or whether it was a valid source, there was no motivation to participate.

#### Overwhelming Amount of Information Online

Participants also felt there was an overwhelming amount of information available online. They felt that they needed a reason to seek out one central resource, and potentially this network could be a place to get the information they desired. Participants said that reading about someone else’s complaints or bad days would negatively influence their participation. They explained that hearing these stories would “stress me out,” “make me feel worse,” or “cause me to think that this could also happen to me.”

#### Competing Influences and Agendas From Pharmaceutical Companies

One consideration that came out of the discussions was who might be driving the discussions and information shared. Although most participants indicated they were comfortable with the CCFA as the “host” for this type of research network and patient portal, there were concerns about the potential influences of pharmaceutical partners. Comments shared by participants highlighted skepticism and concerns that these partners would put profit before patients’ interests if companies could use their data for profit.

#### Data Security and Confidentiality Concerns

The level of data security and confidentiality were highly discussed issues. Participants said most people like them would not want to put their personal health information online, particularly if they had no idea who was going to have access to it. There were also concerns about someone “trolling” for their information.

#### Time Burden and Commitment to Participate

The perceived effort and commitment required for logging in and participating in discussions or to answer survey questions was a common concern for participants. They felt that a daily commitment to enter data was too much effort, but recognized that something less frequent (ie, entering data once a month) would be less useful as IBD is a condition that changes quickly.

### Theme 3: Engagement and Collaboration

When participants were asked about how to ensure active engagement and about what they would advise for building an impactful PPRN, their answers were remarkably homogenous. In general, participants wanted a true partnership and to feel like patients were equal partners in every phase of building the PPRN. They wanted to be a part of open discussions and information exchange with doctors and other participants including health care team members and other patient participants. Participants also explained that they were more likely to sustain long-term participation and involvement if they felt invested in the PPRN’s products and outcomes. In addition, knowing “real” patients had a role in the PPRN’s development made it more appealing to potential participants. Receiving information about additional resources and opportunities from the PPRN via their doctor made patients feel “important” and “like we matter.” A personal invitation with their doctor’s signature would make it seem like a “more special” opportunity. Likewise, regular check-ins in the form of brief email messages or short questions that required some kind of reply or response indicated that someone was paying attention and interested in the patient.

The theme of engagement and collaboration also extended to other participants as well as families and friends. One of the suggestions that emerged from discussions was to frame the PPRN as a group for support and information. Participants wanted to see and feel like they were not “the only one suffering” with IBD. For them, there was also comfort hearing and receiving information in a group setting as it felt “more open and honest.” They also suggested building in learning opportunities for both families and friends to be a part of the process as both were considered key support systems for patients dealing with IBD.

Participants felt it was vital to make sure they were also involved in activities beyond development and data collection. Participants felt strongly that they wanted to be included in the end process. For them, this meant making sure that the “loop was closed” with participants getting some kind of information and feedback. They explained there was frustration from waiting around to see whatever happened with the data collected for research. The overwhelming sentiment was “do not just collect the information and not do anything with it.” Accordingly, if a participant took the time to answer questions or give their information, they wanted to know what happened with the data. Participants felt there needs to be some kind of reporting back to show how the information was being used, and whether or not it was going to be used for a bigger purpose. Ultimately, participants felt successful strategies should involve participatory approaches with patients collaborating throughout the process to identify priorities and define opportunities for support and information. Please see Table 3 for illustrative quotes from participants on their advice for building an impactful PPRN.

**TABLE 3:**
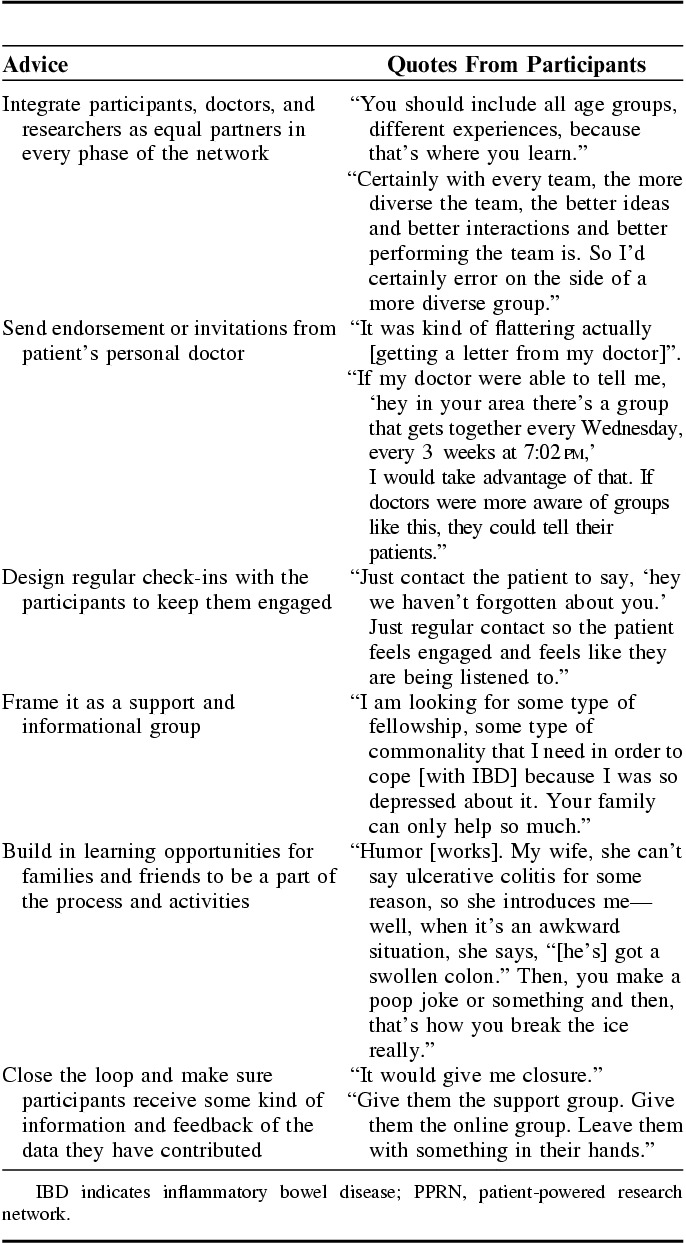
Advice for Building an Impactful PPRN: Illustrative Quotes From Participants

### Theme 4: Customizable Patient Portal Features and Functionalities

Overall, participants indicated they would be more likely to track their health using wearable devices and smartphone applications during flare-ups or when feeling stressed. Some also mentioned the value of using patient-generated health data to track how they were doing and how their body was feeling, particularly given the fact that many only saw their doctors once a year. They were very interested in being able to customize or change features in the patient portal to meet their individual needs. It was particularly important to be able to track and trend health status and symptoms over time, especially between doctor visits, and to have access to personal health records within the portal. Every participant interviewed desired information in the PPRN portal to help with tracking their IBD.

#### Portal Functionalities Related to Tracking

For most participants, tracking nutrition, physical activity, symptoms, and other lifestyle components like sleep was already part of their usual process. However, few had done it formally through wearable devices, smartphone applications, or a portal. In general, they liked the idea of having a systematic way to capture these types of information, but described the process to be “time consuming,” “a hassle,” “expensive,” or “hard to understand.” Even though some indicated tracking devices/apps were more applicable for younger patients, there was interest and willingness among all participants to try out these “new gadgets.”

**FIGURE 1:**
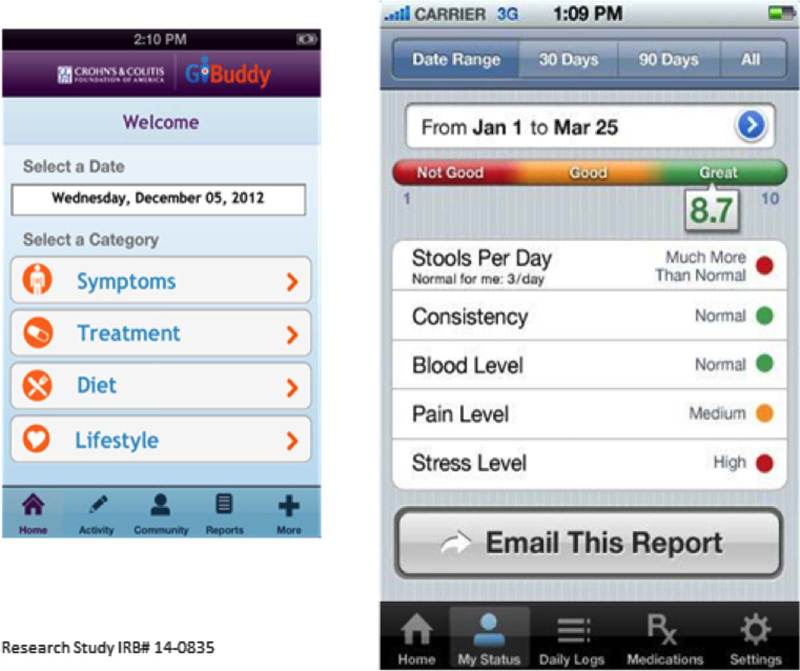
Example of a mockup of the tracking symptoms and quality of life screenshot reviewed during interviews.

When participants were asked to review different tracking screenshots (Fig. 1), there were varying levels of interest and enthusiasm depending on the topic. For example, participants said having the ability to track quality of life and sleep were less important because these were 2 subjective areas with potentially high variability from week to week (ie, holiday seasons, busy work week). However, tracking nutrition and physical activity levels was considered more important as they were described as more directly affecting IBD. The ability to track their IBD had the highest appeal among participants. Participants talked about the benefits of getting a quick glimpse about how they were doing, the usefulness of a stoplight motif color scheme which was universally understood, and the value of having all of the information in one place to assist with better communication with their providers. Lastly, participants said that having a patient portal with functions to access personal records like laboratory results from electronic health records would substantially help support their ability to track their IBD.

#### Tailoring and Customization of the Portal

**FIGURE 2:**
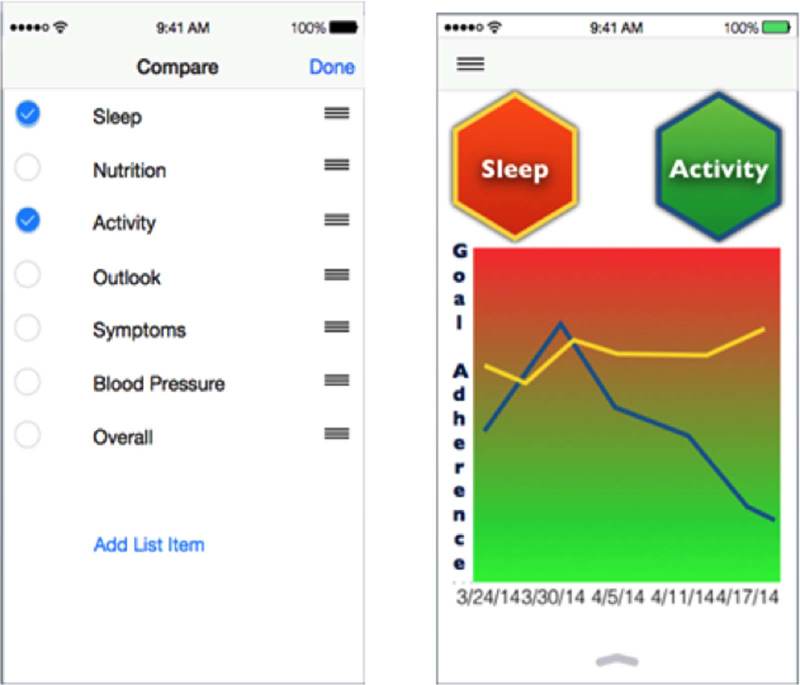
Example of the mockup of the “tailoring what you compare” screenshot reviewed during interviews.

A strong theme from the interviews was that the PPRN patient portal needed to be customizable for each patient given the uniqueness of IBDs. Participants emphasized the importance of being able to tailor the way data are viewed over time, and most wanted the option to pick and choose which data elements to compare over time. They also indicated tracking data over time would be most useful when a participant was feeling sick. During these flare-ups, participants wanted to acquire as much information as possible. Therefore, having the ability to pull the data they wanted quickly was extremely valuable. There was also a sense that tailoring allows participants to have only the information they wanted and needed in a format that was easy to use. As one participant shared, “whatever is created should not require a great deal of effort for the user. We only want to press a few buttons.” When presented with an example of a tailoring screenshot (Fig. 2), participants were not able to automatically see the value of how the two activities were providing useful information. They supported the concept of participants being able to select the activities of higher priority to them, but wanted more information and context directly related to the data display.

#### Individual Trends Over Time

The importance of showing individual trends over time was seen as an imperative feature for a PPRN portal. They also suggested having an option to trend ‘symptoms and treatment’ and ‘symptoms and medication’ as both were considered of high interest. Another suggestion was adding a feature that allows patients to click on specific data points on a line graph to get in-depth feedback about values, events, and other contextual items that could have affected that data points. Overall, the majority of participants said it would be extremely useful to visually see how they were doing over time. Most felt having data trends represented over the last year was ideal. Likewise, participants also explained that having their individual information trended over time was more useful than comparing themselves with others as every individual’s condition was uniquely different.

## DISCUSSION

In summary, participants desire the CCFA Partners PPRN to be a central resource for reliable and unbiased information, to provide a forum for social support, and to offer information about how traditional and alternative medicine therapies may differ in terms of symptom management and outcomes. Participants were motivated to participate in PPRNs to help other patients with IBD but also to contribute toward finding a cure and preventing IBD in others. These findings are similar to those from other online research communities^2^,^4^–^6^ but our findings provide additional specifics which help to operationalize engagement and patient-centeredness within the CCFA Partners PPRN. For the portal, participants prefer customizable, easy-to-use, data displays that can integrate different sources of data (electronic health record data, lifestyle data from smartphone applications and wearable devices/sensors, and patient-reported outcomes). Transparency about how their research contributions are being used and by whom, and data security and privacy were felt to be paramount to providing patient-generated data for research. The preferences for the features and functionalities for PPRN technologies can provide a starting point for future PPRN portals as these are newer concepts.

### Limitations

Our study has several important limitations. Although we purposefully sampled to ensure an informative sample of patients with IBD, we acknowledge that these results may not characterize all patients’ preferences and needs for what a PPRN should offer participants. Although we aimed to enroll more minority patients, we were not successful. This is a reflection of the challenges that other national IBD studies have also experienced. In addition, most of the participants had long-standing IBD so the perspectives of patients who are newly diagnosed could be different.

## CONCLUSIONS

Partnering with patients for the development of the CCFA Partners PPRN was critical for understanding the various needs and preferences of IBD patients as well as for shaping priorities for the network and engagement strategies. Informing the design of the patient portal through this qualitative study was also critical to develop patient-centered designs and meaningful features to facilitate participation in research.

## Supplementary Material

SUPPLEMENTARY MATERIAL

Supplemental Digital Content is available for this article. Direct URL citations appear in the printed text and are provided in the HTML and PDF versions of this article on the journal's Website, www.lww-medicalcare.com.
