# Supplementary material for: Crohn’s and Colitis Foundation of America Partners Patient-Powered Research Network: Patient Perspectives on Facilitators and Barriers to Building an Impactful Patient-Powered Research Network
Source: Med Care. 2018 Sep 13;56(10 Suppl 1):S33–40. doi: 10.1097/MLR.0000000000000771 (PMC6143211; doi:10.1097/MLR.0000000000000771)
Supplement: SUPPLEMENTARY MATERIAL [file mlr-56-s33-s001.docx]

| **Supplemental Table 1. Example of Topics in the Interview Guide for Focus Groups & Individual Interviews on Strategies for a Patient-Powered Research Network** |
| --- |
| - What makes it easier/harder to control or manage your IBD? - Who would you and patients like you trust if they gave you information about IBD? - What do you want to know most about the disease? - What are some reasons you would/would not want to participate in this network? - If this organization could provide any resources you needed to manage your IBD, what would be most helpful to you? - What do you think the network could do to help patients with IBD better understand the opportunities and benefits of participating? - How might you use something like this? What would make you want to use it? - **If you could change one thing about this, whether it is major or minor, what would be at the top of the to-do list?** - What types of items would you want on your summary snapshot that is not included in this slide? What would you want for your health summary? - What would it take for you to use a tool, like a website or app, that can help you better manage your IBD? |
